# Assessment of Personality Traits Influencing the Performance of Men in Team Sports in Terms of the Big Five

**DOI:** 10.3389/fpsyg.2021.679724

**Published:** 2021-05-21

**Authors:** Paweł Piepiora

**Affiliations:** Department of Sports Didactics, Faculty of Physical Education and Sports, University School of Physical Education in Wrocław, Wrocław, Poland

**Keywords:** sport psychology, personality, team sports, championship, assessment

## Abstract

The purpose of this article is to define the perspective from which a coach should analyze and evaluate personality traits that influence sports performance in team sports. The subjects of the research are Polish players (*N* = 300) in senior age (20–29 years) from 10 team sports (each *n* = 30). A sample of champions (*n* = 13) was selected from the study population, and the Big Five model was applied to examine their personality with the use of the NEO Five-Factor Inventory questionnaire. Statistical analyses were performed with the IBM SPSS Statistics software, version 25. The study revealed statistically significant differences between team sports in four personality traits: neuroticism, extraversion, agreeableness, and conscientiousness. Champions of team sports were characterized by a lower level of neuroticism, a higher level of extraversion, and openness to experiences in relation to other sportsmen. It was also confirmed that the personality traits distribution levels depend on the sport discipline. Therefore, an important role must be assigned to those mental training techniques that favor emotional balance, team communication, and tactical thinking skills and are manifested in triggering start-up readiness.

## Introduction

Sports competition is related to an enormous mental burden. Sportsmen must publicly demonstrate their superiority over other sportsmen. It is a media test of their training level. Since the physical skills of sportsmen are often of a similar level, it is assumed that the decisive factor in winning is their personality. Research on personality in sport is extremely popular because it is useful in diagnosing the psychological image of individual sportsmen. Thanks to this, a psychologist can determine the problems that a given sportsperson must deal with. The personality diagnosis allows for the definition of the image of the good, desired, strong, and weak features of a given sportsperson (Piepiora, 2020). Such information is essential for coaches because this way they can guide their charges in an appropriate and most beneficial way. Moreover, the coach’s lack of knowledge about the specificity of personality characteristics and structure of representatives of various sports may adversely affect the development of the sports of their charges and manifest itself in artificial and ineffective activities.

Today’s sport and the ever-increasing demand placed on athletes make all those factors that can help improve the results essential for success. Classified sport is a people’s activity aimed at shaping their personality and mental, physical, and motor excellence, enabling the achievement of sports successes (appropriate for gender, age, and sport discipline). Sports results obtained during the competition and classified by institutions established for this purpose are the measure of excellence. In sport, people always strive to present the maximum of their sporting possibilities; however, the goal of self-improvement is always striving for one’s own perfection, and not only for showing superiority over competitors. Therefore, in self-improvement, the balance between the values of mind and body should never be disturbed. This could be achieved through developing an attitude of cognitive inquisitiveness by using self-empowering and creative methods in the preparation process (Naglak, 1999). Overcoming the barriers set for oneself is the main goal of sports competition because there is no greater reward for an athlete than the act of self-realization in sports combat. One will not cope with that task when the essence of sport is limited to winning competitors. Setting such a shallow goal for an athlete means that the content of the athlete’s preparation for participation in competition is limited only to training perfection in the use of sports techniques and the development of fitness abilities that should be presented during the fight. Wherever only the result counts, other values, such as character training, kindness toward others, and moral virtue, fall into the background. The principle that says sports success is never solely the result of muscle performance should be treated seriously. Success is born and developed where a wise, talented athlete with an extraordinary personality, and well-educated trainer, work together (Lipiec, 1988). That is why athletes are distinguished from people practicing amateur forms of sport, and from people who do not train, by undertaking extreme physical effort, taking into account a high risk of physical injury, tolerating emotional stress in social exposure, maximizing the level of efficiency, achieving long-term goals, and maintaining motivation for high achievements and the ability to postpone gratification (Hardy, 1999; Lazarus, 2000).

The issue of athletes’ personality has been intensively studied since the 1980s in terms of the PEN model and other biological dimensions of personality (Eysenck, 1981, 1985; Guiard, 1981). It was found that the personality of athletes influences their involvement in professional sports. Athletes are less neurotic and more extroverted than nontraining people. They show a low level of anxiety, and they highly rate the psychoticism-superego variable (Eysenck et al., 1982). In addition, there are differences in personality between athletes due to the type of undertaken activity – team sports vs. individual sports (Clingman and Hiliard, 1987; Garland and Barry, 1990) – as well as regarding the sports achievements and the division into classified and unclassified sports (Vealey, 1989). Factors that have been used to explain this profile include assertiveness, thrill seeking, competitiveness, and behavior control (Goldberg and Rosolack, 1994).

Further studies on personality in sport in terms of the five-factor personality model known as the Big Five (McCrae and Costa, 2003) show that physically active people differ in conscientiousness from those who do not train (Mirzaei et al., 2013; Allen and Laborde, 2014). Through physical activity, people are able to direct their life goals and be ambitious in life. It has also been proven that sportsmen who train professionally are distinguished from physically active and nontraining people with a higher level of extraversion and conscientiousness, and a lower index of neuroticism (Allen et al., 2011, 2013; Piepiora and Witkowski, 2018; Piepiora, 2019). Hence, sportsmen are distinguished by the quality and quantity of social interactions as well as the level of activity, energy, and the ability to experience positive emotions. Therefore, sportsmen like to surround themselves with people with a positive attitude. In addition, they are well organized, persistent, and motivated in activities aimed at achieving the intended goal. Sportsmen are also less prone to experience negative emotions and less susceptible to psychological stress. These differences are visible in the level of anxiety, aggressive hostility, tendency to depression, impulsiveness, oversensitivity, or shyness due to involvement in sport. In turn, openness to experience and agreeableness were on a similar level among people representing the area of physical culture. Features, such as trust, straightforwardness, altruism, submissiveness, modesty, tendency to be emotional, imagination, aesthetics, affection, ability to act, idealism, and valence, do not distinguish sportsmen from physically active people.

However, it cannot be unequivocally stated that sports activity can have a beneficial effect in reducing anxiety and depression (Hill et al., 2010). On the other hand, the possible utility of behavior modification in improving athletic performance due to individual and team sports is noted (Nia and Besharat, 2010; Ilyasi and Salehian, 2011). It can be concluded that athletes are characterized by higher emotional stability, extraversion, and responsibility than non-athletes. On the other hand, openness to experience according to the Costa and McCrae (2007) model and the dimension of psychoticism from Eysenck’s (1981) model do not seem to be associated with physical activity.

It is also important that higher-class and successful sportsmen are less neurotic and more extroverted, open-minded, agreeable, and conscientious than the rest of the sportsmen without significant results (Kajtna et al., 2004; Kim et al., 2018; Steca et al., 2018). This indicates that the low level of neuroticism and the remaining high levels of personality traits can be beneficial for sportsmen – since they distinguish the champions from the rest of the sportsmen. Such results were confirmed in the group of martial arts (Piepiora and Witkowski, 2020a) and in the group of individual sports (Piepiora, 2019). Therefore, this study aimed to assess personality traits affecting men’s performance in team sports.

## Materials And Methods

### Research Group

The research was conducted between October 1, 2015, and September 30, 2019. The subjects of the research are players (*N* = 300) from 10 team sports, selected on purpose non-randomly from the Polish population of men training competitive sports. Such a selection of respondents was dependent on the voluntary willingness of male athletes to participate in the study and their high position in Polish team sports. Moreover, respondents had to be of senior sports age: between the ages of 20 and 29. They had to have at least the second sports class, sports experience – at least starting from a cadet age (14 years old), professional training – minimum 3 years, a current competition license, their coach’s consent to participate in the study, and documented sports achievements at various levels of competition (national, continental, and world). Athletes who refused to participate in the study were excluded from the selection of leading Polish team sports athletes. Therefore, it was possible to collect 10 homogeneous samples (each *n* = 30) from the following sports: American football, basketball, beach volleyball, floorball, football, futsal, handball, indoor volleyball, rugby, and ultimate frisbee. Then, players with international sports achievements were selected from the entire population of surveyed players and qualified to the champions sample (*n* = 13). The criterion for selecting nonrandom, purposeful respondents to the champions sample was dictated by their documented success (first, second, or third place) at international competitions in given sports disciplines. There were 2 beach volleyball players, 2 floorball players, 2 futsal players, and 7 indoor volleyball players. The remaining sportsmen (*n* = 287) are those with only successes on a Polish (national) level. The author did not receive consent from coaches and players for the use of detailed variables in the study, such as age, seniority in sports, and sports achievement, as the detailed data would compromise the anonymity of the study participants. Moreover, it should be noted that the personality of the respondents and their achievements were verified once, during the study time. The studies on the respondents were not repeated within the span of 4 years (the overall length of the research), and the records of achieved successes were not modified in relation to the further careers of the researched athletes.

### Methods

A five-factor personality model, known as the Big Five, was used to examine the players’ personality. It includes five main features, factors, or dimensions: neuroticism, extraversion, openness to experience, agreeableness, and conscientiousness, which allow for their separate classification, forming NEOAC, OCEAN, or CANOE acronyms. Upon characterizing the Big Five, one should pay attention to several important aspects of character traits in terms of Costa and McCrae (2007) perspective. These features characterize the so-called “normal personality,” although their extreme severity may contribute to the development of behavioral disorders and psychosomatic diseases. In this sense, a simple clinical interpretation should not be adopted in relation to the Big Five model. Dimensions are not of the classical type, but are continuous, and – like other psychological properties – have a normal distribution in the population. The extreme poles of traits are associated with the positive and negative trends in behavior, both for the social environment and for a given individual. Therefore, each personality feature has its advantages and disadvantages. The features meet the criteria required to name the basic dimensions of personality. The formal criteria for the characteristics of the five-factor model of personality were formulated by Costa and McCrae during the discussion on the basic dimensions of personality (Eysenck, 1991, 1992; Costa and McCrae, 1992a,b).

For this purpose, the NEO Five-Factor Inventory (NEO-FFI) personality questionnaire was used due to the study time acceptable for the competitive players in voluntary research. For this study, the NEOAC acronym was adopted. The questionnaire consists of 60 self-descriptive statements, the truthfulness of which in relation to themselves was assessed by the respondents on a five-point scale, varying from the statement “definitely not” to the statement “definitely yes.” NEO-FFI has sten norms for five age groups (15–19, 20–29, 30–39, 40–49, and 50–80), developed separately for women and men on the basis of large population samples. In addition, it is internally compatible. The obtained results allow for a full description of the respondents’ personality in terms of the Big Five and for forecasting their adaptation possibilities to the professional sport environment (Costa and McCrae, 2007).

Statistical analyses were performed using IBM SPSS Statistics, version 25. A series of one-way ANOVAs were performed. Welch’s correction was applied in case of breaking the assumption of homogeneity of the variance. In the case of unequal groups, breaking this assumption is the most problematic from the point of view of drawing conclusions; therefore, this correction is necessary. Parameters were estimated using the bootstrapping method with a sampling set at 5,000 and 95% confidence intervals. On the other hand, when the assumption of homogeneity of variance was broken, Games-Howell tests were used in *post-hoc* analyses. And if the assumption of homogeneity of variance was met, Tukey’s tests were used. Additionally, due to the multiple comparisons made within each sport category, it was decided to adopt the Bonferroni correction for the significance level. In each sports category, five one-way ANOVAs were performed, and the level of statistical significance for the ANOVAs was calculated as *α* = 0.01.

### Procedure

Each tested player agreed to participate in the research after getting acquainted with the information on the objectives and principles of carrying out, expected effects, and possible benefits for the study participants. The respondents also familiarized themselves with the risk associated with undergoing the study, indicating the mode and possibility of withdrawing from participation in the study at each stage. Moreover, the respondents were informed that they had the possibility to ask questions and obtain answers to them. All respondents consented to the use of the results of the NEO-FFI research, but did not consent to the use of data that would compromise their anonymity. Competitors have this stipulated in their contracts. The respondents had 1 h to respond to the statements of the NEO-FFI personality questionnaire. The research was carried out in groups of up to 30 people. After the research work was completed, the participants’ data were coded.

All respondents gave their informed consent to participate in the study. All procedures carried out in the human trials were in accordance with the ethical standards of the institution and/or the National Research Commission and the 1964 Helsinki Declaration and its subsequent amendments. The project received a positive opinion (number 20/2019) from the Senate Committee on Ethics of Scientific Research at the University School of Physical Education in Wrocław.

## Results

The obtained results showed significant differences between individual disciplines in the following traits: neuroticism, extraversion, agreeableness, and conscientiousness. The strongest effect was again observed for neuroticism – around 18% of the explained variance. In the analysis of differences in conscientiousness, about 14% of the explained variability was observed. On the other hand, in extraversion and conscientiousness, the differences in the effects amounted to approximately 7 and 6% of the explained variance, respectively.

The highest level of neuroticism was noted among Ultimate Frisbee players, and it was statistically significantly different from all other team sports disciplines. In addition, American football players were also characterized by higher neuroticism than rugby and football players. Floorball players also had a higher neuroticism index than rugby and football players.

**Figure 1:**
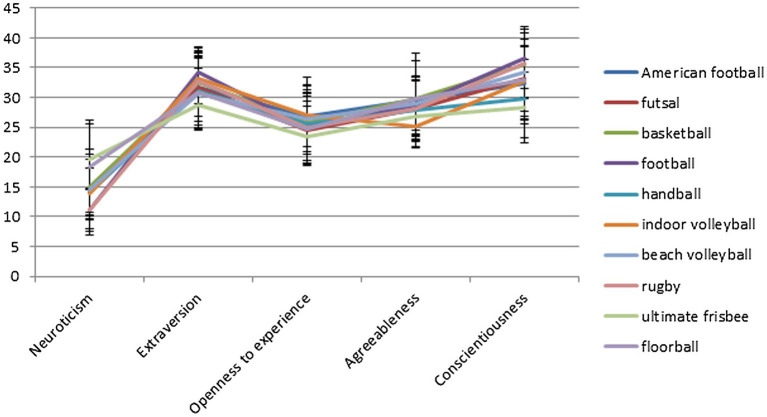
Line chart of personality profiles of team sports players.

*Post-hoc* tests in the extraversion dimension revealed significant differences, with ultimate frisbee players showing a significantly lower intensity of this trait in relation to football, indoor volleyball, and rugby players. Ultimate frisbee players also showed a lower level of conscientiousness than basketball, football, beach volleyball, and rugby players. Additionally, handball players showed significantly lower conscientiousness than basketball players and rugby players. One significant difference in agreeableness was found between volleyball players and basketball players, with the former having a lower agreeableness marker than the latter. The coefficient values of the performed models of one-way ANOVAs are included in Table 1, and the whole is shown in Figure 1.

**Table 1:** Analysis of differences between team sports disciplines for individual personality traits.

Disciplines	Personality traits									
	Neuroticism		Extraversion		Openness to experience		Agreeableness		Conscientiousness	
	*M*	*SD*	*M*	*SD*	*M*	*SD*	*M*	*SD*	*M*	*SD*
American football (*n* = 30)	14.97	4.58	31.67	6.74	26.87	5.08	29.63	7.81	32.33	6.14
Futsal (*n* = 0)	14.07	7.20	31.73	5.78	24.60	6.01	28.10	5.54	33.23	7.57
Basketball (*n* = 30)	15.03	5.54	32.63	5.75	25.87	5.38	29.87	6.21	35.57	5.82
Football (*n* = 30)	11.23	3.35	34.27	3.59	25.13	5.64	28.70	4.77	36.67	5.17
Handball (*n* = 30)	14.17	4.01	31.07	6.45	25.57	6.67	27.83	5.09	29.90	6.58
Indoor volleyball (*n* = 30)	13.90	4.26	33.30	4.26	27.03	3.30	25.17	3.71	32.77	5.84
Beach volleyball (*n* = 30)	14.47	6.84	30.63	6.19	26.40	7.06	28.93	4.43	34.27	6.58
Rugby (*n* = 30)	11.23	3.35	32.83	4.25	24.87	5.83	28.10	5.30	35.77	5.73
Ultimate frisbee (*n* = 30)	19.63	4.86	28.83	3.43	23.50	4.81	26.87	3.75	28.33	5.97
Floorball (*n* = 30)	18.40	7.73	30.83	5.81	24.70	3.90	29.80	6.37	33.03	6.68
*F*	9.90^*^		2.53^*^		1.24^*^		2.17^*^		5.21	
*df*	9; 117.74		9; 117.78		9; 117.75		9; 117.87		9; 290	
*p*	<0.001		0.008		0.078		0.003		<0.001	
*η*^2^	0.18		0.07		0.04		0.06		0.14	

* *Correction for heterogeneity of variance.*

**Figure 2:**
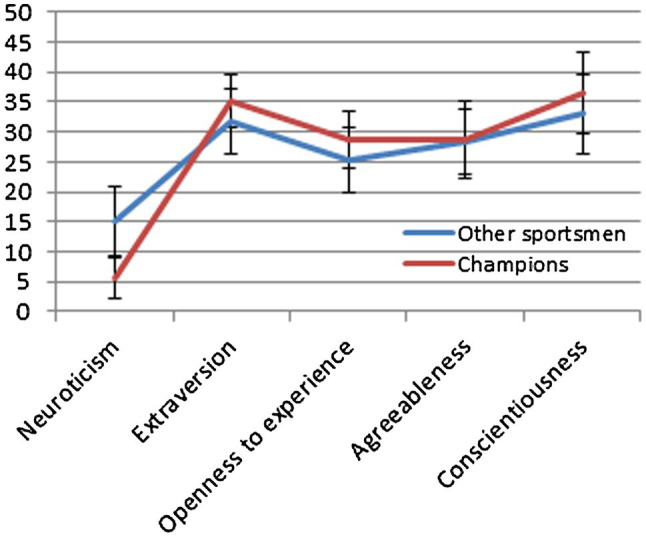
Line chart of personality profiles of champions and other team sportsmen.

The same analyses were then repeated between the champions and other team sports players. Significant differences were observed for neuroticism and extraversion. The difference in openness to experience was not statistically significant after taking into account Bonferroni correction. However, due to the moderately strong effect, it was also described. Again, a very strong effect was seen in the differences in neuroticism, and moderately strong effects were found for extraversion and openness to experience. Team sports champions were distinguished by a lower level of neuroticism, a higher level of extraversion, and openness to experience in relation to other players. The detailed results are presented in Table 2 and Figure 2.

**Table 2:** Analysis of differences between champions and other team sportsmen in the intensity of individual personality traits.

Variables	Other sportsmen (*n* = 287)		Champions (*n* = 13)				
	*M*	*SD*	*M*	*SD*	*t*	*p*	Cohen’s *d*
Neuroticism	15.12	5.64	5.69	3.35	5.98	<0.001	1.69
Extraversion	31.62	5.47	35.23	4.40	−2.34	0.003	0.66
Openness to experience	25.31	5.50	28.54	4.74	−2.08	0.013	0.59
Agreeableness	28.28	5.50	28.69	6.33	−0.26	0.794	0.07
Conscientiousness	33.03	6.59	36.54	6.70	−1.87	0.058	0.53

## Discussion

Among the studied population of team sports players, significant differences were found between particular sports disciplines, i.e., in neuroticism, extraversion, agreeableness, and conscientiousness. It was found that there are differences in the intensity of individual personality traits between sports disciplines within team sports. This indicates the existence of differences in the personality of players depending on their sports discipline. The obtained data indicate significant effects of sport on the personality shaping of the assessed players and confirm the reports of other authors (Chirivella and Martinez, 1994; Kajtna et al., 2004; Tok, 2013; Kang et al., 2016; McEwan et al., 2019; Piepiora et al., 2020). Sports activity shapes the personality, and the formed personality traits have an impact on taking solutions in the starting situation. It should be related to the specificity of sports competition and the slightly different psychological requirements that sports disciplines impose on competitors. However, these dissonances have not been noted in openness to experience. Taking into account the specificity of acceptable contact in team sports, it was assumed that the lack of differences in openness to experience was revealed primarily in the divergent thinking and creativity of players (Costa and McCrae, 2007). Moreover, the previous experiences of the surveyed players from the earlier periods of their sports careers and the impact of many years of sports training on possible modifications of personality traits should be taken into account. Apart from the influence of the coach and other entities from the players’ closest social environment. Therefore, social and cultural factors must also be taken into account.

In the second stage of research, it was discovered that the champions of team sports were distinguished from other players by a lower level of neuroticism and a higher level of extraversion and openness to experience. The obtained empirical evidence confirms the claims of other researchers (Tutko and Ogilvie, 1966; Schur et al., 1977; Garland and Barry, 1990; Lerner and Locke, 1995; Carver and Scheider, 1998; Turk et al., 2001; Gardner and Moore, 2006, 2007). Personality traits can be used as predictors of sports performance. It was presumed that the intensity level of neuroticism, extraversion, and openness to experience in players of team sports may affect the sports performance.

The general profile of sportsmen in terms of the Big Five is low neuroticism, high extraversion, and conscientiousness, as well as average openness to experience and agreeableness (Backmand et al., 2003; McKelvie et al., 2003; Anghel et al., 2009). In the obtained data, it was observed that the team sports champions are characterized by lower neuroticism and higher extraversion and openness to experience. The other factors did not differ statistically from the rest of the players. These results are confirmed by known studies (Piedmont et al., 1999; Shrivastaval et al., 2010; Kim et al., 2018). However, referring to the Polish norms of NEO-FFI interpretation (Costa and McCrae, 2007), all the respondents – champions and other team games players – are distinguished by high rates of extraversion and conscientiousness as well as average rates of openness to experience and agreeableness. The difference between the champions and other athletes is only in the dimension of neuroticism. Champions are characterized by low neuroticism in relation to other team games players, who achieved an average result in this dimension.

Team sports are characterized by conflict resolution methods consisting in demonstrating, in accordance with the adopted rules, superiority over the opponent in a certain range, which leads to gain temporary access to the resources. The will to dominate concerns actions or undertakings that are characterized by a lack of intention to destroy an opponent. The advantage mainly relates to higher performance in the game. That is why champions of team sports are characterized by a low level of neuroticism manifested in the pursuit of direct contact (Piepiora and Witkowski, 2020b), a high level of extraversion manifested in team communication (Ilyasi and Salehian, 2011), and a high level of openness to experiences manifested in thinking divergence and creativity of athletes (Costa and McCrae, 2007), which was not shown by non-champions in team sports.

It should be noted that these findings confirm the role of passion in sport and set new directions for research. A high level of sportsmanship and an autonomous personality orientation lead to a harmonious passion, while a high level of sports valuation and controlled personality orientation favor an obsessive passion (Vallerand et al., 2003, 2008). What makes a champion depends not only on good genetics, innate talent, and physical strength but also on mental abilities and personality traits (Tomar and Singh, 2012; Murnieks et al., 2014).

Accordingly, the forward-looking assessment of personality traits influencing performance in team sports in terms of the Big Five refers to neuroticism, extraversion, and openness to experience. Therefore, in the mental preparation of a sportsperson, particular attention should be paid to those aspects of psychological preparation that are adequate to the components of the above-mentioned dimensions. In team sports games, high emotional stability reflects the confidence and mental resilience of players. High extraversion manifests itself in interpersonal relations between players and communication in the team. And openness to experience reflects the thinking divergence of players, their creativity, and “reading the game,” manifested by making tactical decisions.

It should be noted that the obtained research results have great application value at the stage of sport selection, training, and sports competition. They can constitute the basis for the development of appropriate practical directives, important in the sports training of high-class players. It is suggested that in the sports selection of high-class players on the national team, the first verification stage should be the distribution level of personality traits. Candidates meeting the criteria of low neuroticism, high extraversion, and openness to experience may be the desired individuals at the mental selection stage. Only in the second stage, physical criteria should be taken into account, i.e., somatic build and motor, technical and tactical predispositions, and the achievements of the contenders.

Here, the strength and limitations of the test should be equally indicated. The research sample was homogeneous in terms of ethnicity, gender, and age range of 20–29 years. Athletes of other nationalities, women, and other age groups were not included. The research was conducted with a large group of respondents from sports disciplines popular in Poland. However, it was not possible to examine the players from all team sports games trained in Poland. The variables were distributed in equal samples. The groups of champions and other athletes were not even, but they were divided into Polish winners with international sports successes and players at the national level. Therefore, the obtained research results can only be reduced to a specific population of athletes. The author does not question the current results with his research results, but only supplements the existing knowledge about personality in sport.

## Conclusion

The conducted research proved that the distribution levels of personality traits depend on the sport discipline. And the champions are distinguished from the remaining team sports players (aged between 20 and 29) by a low level of neuroticism and a high level of extraversion and openness to experience. Therefore, an important role must be assigned to those mental training techniques that promote emotional balance, team communication, and tactical thinking skills, and are manifested in triggering start-up readiness.

## Data Availability Statement

The original contributions presented in the study are included in the article, and further inquiries can be directed to the corresponding author.

## Ethics Statement

The studies involving human participants were reviewed and approved by the Senate Committee on Ethics of Scientific Research at the University School of Physical Education in Wrocław – number 20/2019. The patients/participants provided their written informed consent to participate in this study.

## Author Contributions

The author confirms being the sole contributor of this work and has approved it for publication.

### Conflict of Interest

The author declares that the research was conducted in the absence of any commercial or financial relationships that could be construed as a potential conflict of interest.
